# IgG4-related hypertrophic pachymeningitis presenting with marked dural thickening, widespread white matter changes, and rectus gyrus transdiaphragmatic herniation into the sella turcica: a case report

**DOI:** 10.3389/fimmu.2026.1776836

**Published:** 2026-02-11

**Authors:** Takashi Ogawa, Kazuo Yamashiro, Satoshi Tsutsumi, Isamu Takai, Reika Kiuchi, Yuto Hirata, Ryota Nakamura, Toshiki Nakahara, Masao Watanabe, Nobutaka Hattori, Taku Hatano, Takao Urabe

**Affiliations:** 1 Department of Neurology, Juntendo University Urayasu Hospital, Chiba, Japan; 2 Department of Neurosurgery, Juntendo University Urayasu Hospital, Chiba, Japan; 3 Department of Neurology, Faculty of Medicine, Juntendo University, Tokyo, Japan

**Keywords:** dural thickening, hypertrophic pachymeningitis, IgG4-related disease, *Sella turcica* (rectus gyrus transdiaphragmatic herniation), white matter lesions

## Abstract

**Introduction:**

IgG4-related disease is a clinically significant immune-mediated condition that can involve multiple organs. In the central nervous system, IgG4-related hypertrophic pachymeningitis is characterized by dural thickening, and the resulting mass effect may lead to various neurological deficits and characteristic imaging findings.

**Case description:**

A 54-year-old Japanese man presented with a 6-month history of slowly progressive right-sided visual impairment and visual field loss. Neurological examination revealed no abnormalities other than reduced visual acuity and visual field defects. Cranial magnetic resonance imaging revealed marked dural thickening extensively involving the bilateral frontotemporal regions, widespread frontal white matter lesions, and transdiaphragmatic herniation of the rectus gyrus into the sella turcica. The serum IgG4 level was elevated (429 mg/dL), and a dural biopsy revealed inflammatory cell infiltration with IgG4-positive plasma cells, leading to a diagnosis of IgG4-related hypertrophic pachymeningitis. Systemic evaluation, including laboratory screening and trunk computed tomography, revealed no other organ involvement apart from cervical and hilar lymphadenopathy. The patient responded well to steroid treatment (intravenous methylprednisolone followed by tapered oral prednisolone), with gradual improvement of the dural thickening, white matter lesions, rectus gyrus herniation, and visual field defects over 3 months. The serum IgG4 level decreased to 70.3 mg/dL.

**Discussion:**

This case was characterized by pronounced dural thickening, widespread white matter lesions, and unprecedented rectus gyrus herniation into the sella turcica, a combination of features not previously reported. Neurologists should consider IgG4-related disease in the differential diagnosis of hypertrophic pachymeningitis accompanied by white matter lesions because early recognition and treatment may prevent irreversible neurological damage.

## Introduction

IgG4-related disease (IgG4-RD) is an immune-mediated fibroinflammatory condition characterized by tumefactive lesions, lymphoplasmacytic infiltration, and storiform fibrosis. Although involvement of various organs, including the gastrointestinal tract, is well documented (1, 2), central nervous system (CNS) manifestations—primarily IgG4-related hypertrophic pachymeningitis (IgG4-RHP)—are rare. Notably, isolated CNS involvement is increasingly recognized, with approximately 30% to 50% of patients lacking systemic manifestations at diagnosis (3).

The clinical presentation varies according to the site of dural inflammation, most commonly manifesting as headaches, cranial nerve palsies, and visual disturbances (4). Diagnosis requires a multimodal approach that integrates elevated serum IgG4 levels, characteristic dural thickening on magnetic resonance imaging (MRI), and histopathological confirmation of IgG4-positive plasma cell infiltration with storiform fibrosis (5).

High-dose glucocorticoids remain the first-line therapy; however, relapse during tapering represents a significant clinical challenge. In refractory cases, B-cell depletion therapy has become the standard second-line option (6). More recently, the therapeutic landscape has expanded with the approval of the anti-CD19 antibody inebilizumab, offering a new targeted strategy for preventing disease flares (7).

In this report, we describe a complex case of IgG4-RHP presenting with visual impairment, marked dural thickening, and concomitant white matter lesions. A distinctive feature of this case was the transdiaphragmatic herniation of the rectus gyrus into the sella turcica—a rare neuroradiological finding that highlights the potential for severe mechanical complications in IgG4-RHP and underscores the importance of early diagnostic recognition and intervention.

## Case description

A 54-year-old Japanese man presented with right-sided visual impairment and visual field loss. His medical history included hypertension, and he was a former smoker. Six months before presentation, he developed blurred vision in the right eye, which persisted despite treatment at a local clinic. Four months later, a gradual visual field defect appeared in the temporal region of the right eye.

**Figure 1:**
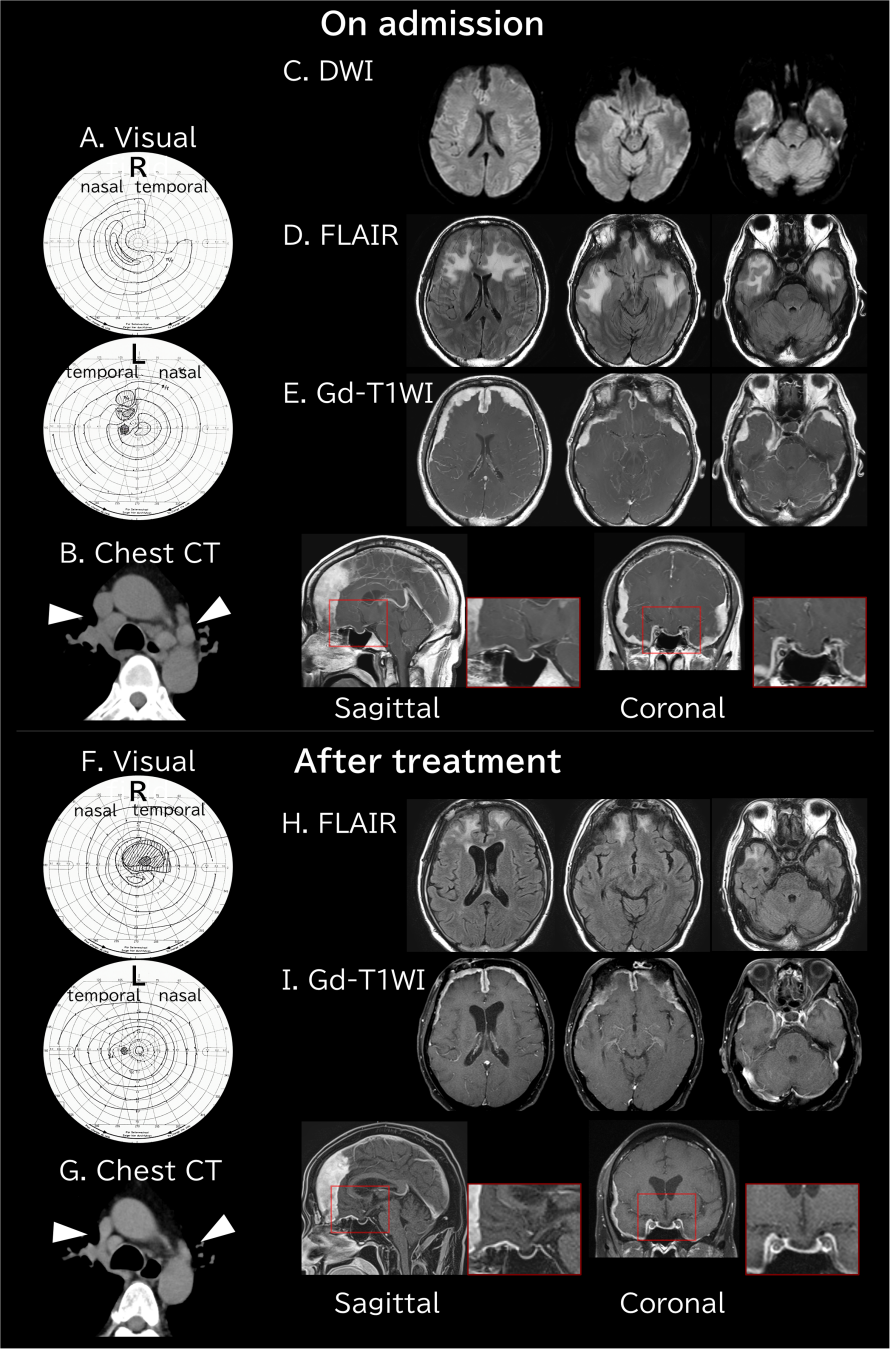
Visual field testing and radiological findings before and after treatment. **(A)** On admission, visual field testing showed loss of the upper temporal quadrant of the right visual field and a scotoma in the left visual field. **(B)** Chest CT revealed hilar lymphadenopathy (white arrowheads). **(C)** DWI showed no obvious diffusion restriction. **(D)** FLAIR images demonstrated widespread high-intensity signals in the anterior white matter. **(E)** Gd-T1WI revealed diffuse dural thickening extending into the frontal and temporal regions, with herniation of the rectus gyrus of the frontal lobe into the sella turcica. **(F)** Three months after steroid treatment, visual field testing showed recovery of the right peripheral visual field and reduction of the left scotoma. **(G)** Follow-up chest CT showed marked regression of the hilar lymphadenopathy. **(H)** The widespread high-intensity white matter signals had decreased, and the dural thickening had markedly improved. **(I)** Correspondingly, herniation of the rectus gyrus also showed improvement. CT, computed tomography; DWI, diffusion-weighted imaging; FLAIR, fluid-attenuated inversion recovery; Gd-T1WI, gadolinium-enhanced T1-weighted imaging; L, left; R, right.

On physical examination, the patient was alert and oriented. Aside from visual symptoms, no focal motor or sensory deficits or other cranial nerve abnormalities were identified. There was swelling of the right cervical lymph nodes, blindness in the upper quadrant of the right eye on the ear side, an enlarged scotoma in the left eye (**Figure 1A**), and markedly reduced visual acuity of 0.02 in the right eye.

Blood tests revealed elevated levels of C-reactive protein (2.5 mg/dL; reference range, <0.3 mg/dL), IgG (2068 mg/dL; reference range, 870–1700 mg/dL), and IgG4 (429 mg/dL; reference range, <135 mg/dL). Tumor markers, including carcinoembryonic antigen, carbohydrate antigen 19-9, prostate-specific antigen, and serum soluble interleukin-2 receptor, were all within normal limits. Serologic tests for collagen vascular diseases—such as anti-nuclear antibody, Sjögren’s syndrome antigen A/B antibodies, and proteinase 3-specific and myeloperoxidase-specific antineutrophil cytoplasmic antibodies—were also negative. The angiotensin-converting enzyme level was unremarkable, and no pituitary dysfunction was detected.

To screen for systemic IgG4-RD, non-contrast computed tomography (CT) of the chest including the neck and of the abdomen including the pelvis was performed. This revealed hilar lymphadenopathy (**Figure 1B**) but no other organ involvement, such as pancreatitis or retroperitoneal fibrosis. Head MRI showed widespread signal changes in the frontal white matter. Gadolinium-enhanced MRI demonstrated a subdural mass lesion extensively involving the bilateral frontotemporal regions. Bilateral compression was present, causing the rectus gyrus to be abnormally displaced downward with transdiaphragmatic herniation into the sella turcica (**Figures 1C–E**). Despite the bitemporal lesions, electroencephalography was not performed because the patient exhibited no clinical seizures. Lumbar puncture was not performed because of the risk of tonsillar herniation, and cerebrospinal fluid could not be obtained during biopsy because of severe adhesion between the subdural lesion and the cerebral cortices, with absence of visible subarachnoid spaces.

**Figure 2:**
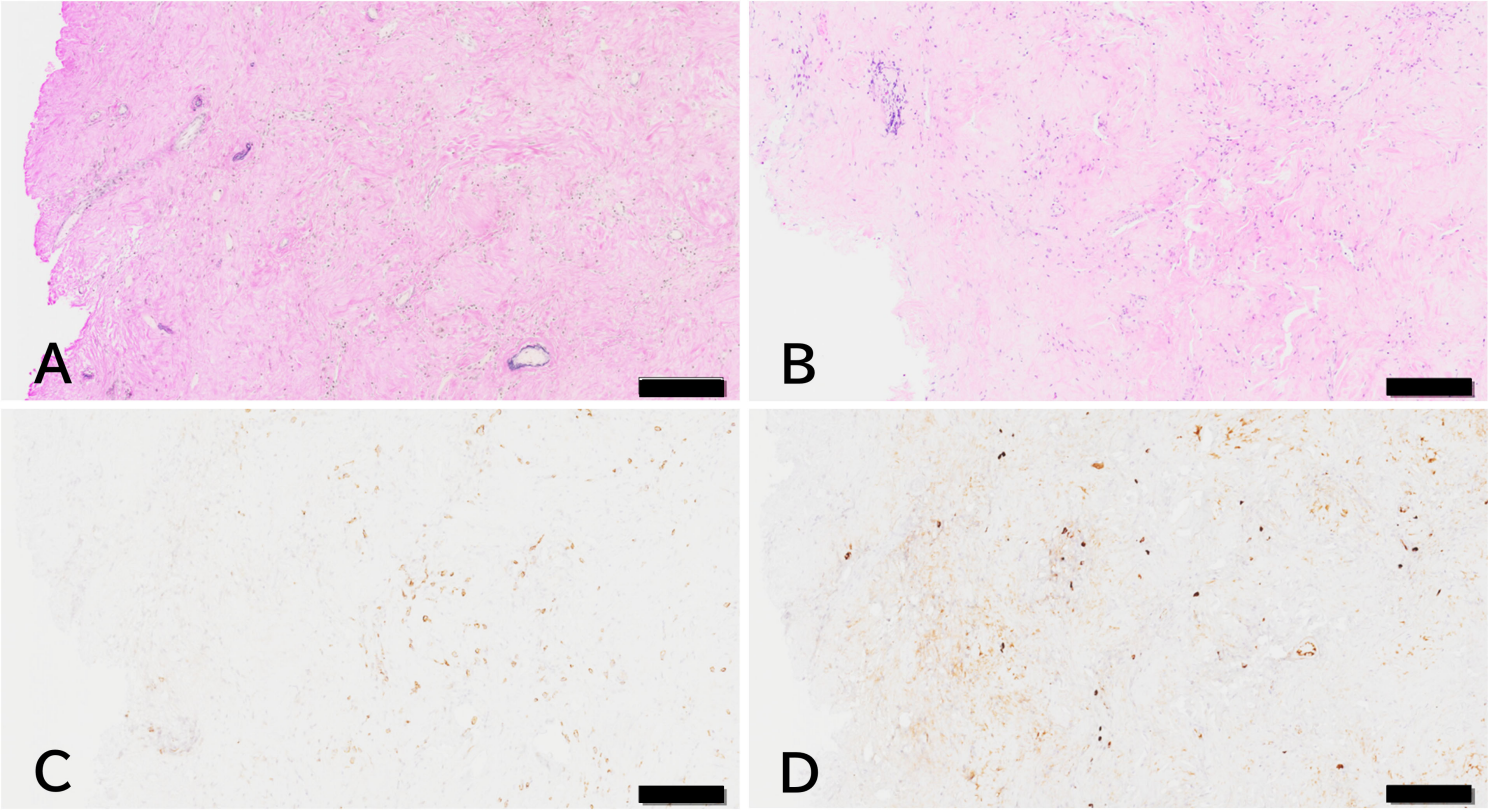
Histological findings of dural biopsy specimen. **(A)** Elastica van Gieson staining showed abundant collagen fibers within the dura. **(B)** Hematoxylin and eosin staining demonstrated inflammatory cell infiltration. **(C)** CD138-positive plasma cell infiltration was observed, and **(D)** approximately half of these cells were IgG4-positive. Scale bars: 200 μm.

A biopsy was performed via a small frontal craniotomy. The lesion appeared whitish, continuous with the overlying dura mater, firmly adherent to the underlying cerebral cortices, elastic-hard in consistency, and relatively avascular. Microscopically, the resected specimens demonstrated markedly thickened dura mater with infiltration by inflammatory cells, approximately half of which stained positively for IgG4 (**Figure 2**).

The patient was diagnosed with IgG4-related hypertrophic pachymeningitis and treated with intravenous methylprednisolone (1000 mg for 3 days). Oral prednisolone was then initiated at 1 mg/kg (60 mg), tapered by 10 mg per week to 20 mg, and subsequently reduced by 5 mg per month.

**Figure 3:**
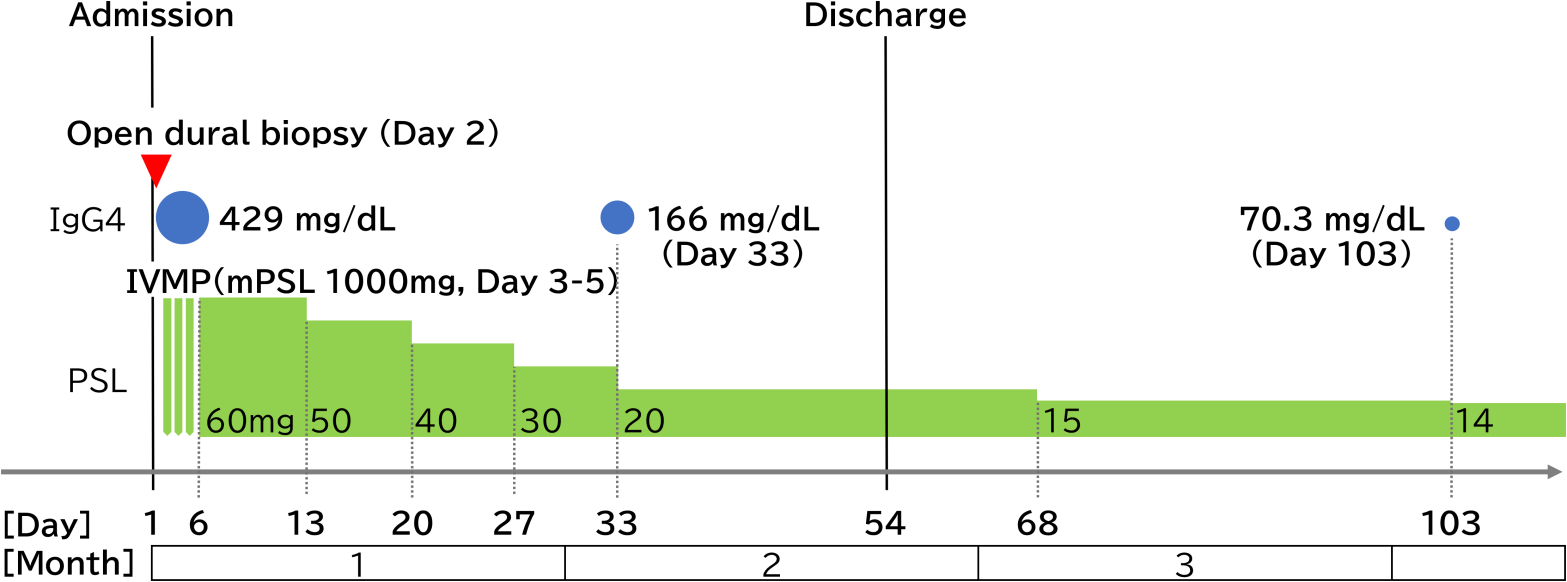
Clinical course from admission to Day 103. The x-axis represents the timeline from admission (Day 1). Following an open dural biopsy on Day 2, IVMP (1000 mg/day) was administered for 3 days starting on Day 3. Oral PSL was initiated at 1 mg/kg (60 mg/day) on Day 6, tapered by 10 mg per week to 20 mg/day, and subsequently reduced by 5 mg per month. Serum IgG4 levels (blue circles) decreased from 429 mg/dL (Day 1) to 166 mg/dL (Day 33) and 70.3 mg/dL (Day 103), paralleling the improvement in visual field testing and MRI findings (see **Figure 1**). IVMP, intravenous methylprednisolone; mPSL, methylprednisolone; PSL, prednisolone.

Three months after treatment initiation (Day 103), the serum IgG4 level had decreased from 429 to 70.3 mg/dL, with an intermediate value of 166 mg/dL on Day 33 (**Figure 3**). The patient was receiving oral prednisolone at 14 mg/day, with a tapering target of 10 mg/day. Given the chronic nature of the disease, maintenance therapy with inebilizumab is under consideration for future management. Peripheral vision had also improved, although a scotoma remained in the right visual field, while the left-sided scotoma had diminished (**Figure 1F**). Follow-up chest CT demonstrated a marked reduction in the size of the previously noted hilar lymphadenopathy (**Figure 1G**). Cranial MRI showed regression of the frontal white matter lesions and thinning of the previously thickened dura mater on gadolinium-enhanced imaging. Although the dural thickening improved after 3 months (**Figures 1H, I**), follow-up cerebrospinal fluid sampling has not been performed to date because the herniation of the rectus gyrus into the sella turcica persisted.

## Discussion

According to the 2020 revised comprehensive diagnostic criteria for IgG4-RD (8), this case was definitively diagnosed on the basis of systemic involvement (cervical and hilar lymph node enlargement), dural thickening, marked elevation of serum IgG4, and biopsy findings demonstrating dural fibrosis with infiltration of IgG4-positive plasma cells. Although the patient presented primarily with neurological symptoms, the identification of hilar lymphadenopathy via systemic CT screening was instrumental in supporting the diagnosis of a multiorgan fibroinflammatory process. Notably, the marked regression of these lymph nodes following glucocorticoid therapy confirmed their involvement in the systemic pathology of IgG4-RD. This underscores the importance of comprehensive systemic screening because identification of subclinical organ involvement can provide critical diagnostic confirmation even in cases that initially appear to represent isolated CNS disease.

Ten cases of IgG4-RHP with significant white matter lesions have been reported to date, including the present case (**Table 1**) (9–17). As detailed in **Table 1**, therapeutic strategies in these cases largely depended on lesion morphology. Cases presenting with focal mass lesions were predominantly managed with surgical resection, often because of an initial suspicion of neoplasms such as meningioma; in these instances, additional steroid therapy was typically not required following successful excision. By contrast, patients with diffuse dural thickening, including the present case, were treated with high-dose glucocorticoids and consistently demonstrated a favorable response. This clinical course supports the hypothesis that the associated white matter lesions represented reversible vasogenic edema caused by impaired venous or lymphatic drainage due to dural inflammation and mechanical compression, rather than permanent ischemic injury (3). Whereas dural biopsy was essential for definitive histological diagnosis, therapeutic surgical resection of the white matter lesions was not indicated because these changes were secondary and reversible, responding appropriately to medical therapy.

**Table 1:** Reported cases of IgG4-related hypertrophic pachymeningitis with significant white matter lesions.

Reference	Age (y), sex	Symptoms and signs	Location of thickened dura	White matter lesions	Primary treatment	Outcome
Kim EH. *J Neurosurg.* 2011 (9)	43, M	Headache, paresis	Left parietal (focal)	Parietal - diffuse	Surgery + Steroids	Good recovery
Li LF. *World Neurosurg.* 2015 (10)	58, F	Motor paresis	Falx cerebri (focal)	Parietal - focal	Surgical resection	No recurrence
Goulam-Houssein S. *Neuroradiol J.* 2018	70, M	Diplopia	Left frontotemporal (focal)	Temporal - diffuse	Surgical resection	Stable
Zhang Z. *Acta Neurol Belg.* 2018 (12)	29, M	Memory disturbance	Left frontal (focal)	Frontal - diffuse	Steroids (PSL)	Good recovery
Chen M. *Am J Med Sci.* 2023 (13)	54, M	Seizure	Right frontal (focal)	Frontal - focal	Surgical resection	Symptom free
Simbaqueba C. *Ann Intern Med.* 2023 (14)	71, M	Facial nerve palsy	Temporoparietal (diffuse)	Temporoparietal - diffuse	Steroids (mPSL)	Good recovery
Zhang Y. *Radiol Case Rep.* 2024 (15)	56, M	Dizziness	Left parietal (focal)	Parietal - focal	Surgical resection	Recovered
Chou ML. *Med Sci.* 2024 (16)	65, F	Seizure	Right frontal (focal)	Frontal - focal	Surgical resection	Seizure free
Fang CY. *Ann Intern Med.* 2024 (17)	67, M	Confusion	Left temporal (focal)	Temporal - focal	Steroids (mPSL)	Good recovery
Present case	54, M	Visual impairments	Bilateral (diffuse)	Bilateral - diffuse	Steroids (mPSL -> PSL)	Improved (residual herniation)

F, female; M, male; mPSL, methylprednisolone; PSL, prednisolone.

Although B-cell depletion therapies such as rituximab or the recently approved inebilizumab were not used in the reviewed cases—likely because of adequate responses to first-line therapy or the timing of the reports—they are now strongly recommended for patients with refractory disease, with frequent relapses, or requiring steroid-sparing regimens to prevent long-term morbidity. The right-sided quadrant hemianopsia and the enlarged left-sided scotoma in this patient can be explained by compression of the optic chiasm. Consistent with prior reports, the patient responded well to steroid therapy, showing marked clinical and radiological improvement.

In addition, MRI demonstrated herniation of the rectus gyrus into the sella turcica without evidence of hypopituitarism. Although cases of rectus gyrus herniation have been described in association with empty sella following pituitary surgery (18, 19), no prior reports have documented transdiaphragmatic herniation caused by chronic downward displacement from dural thickening. Notably, although the dural mass decreased substantially after treatment, the herniated rectus gyrus remained displaced at the 3-month follow-up. This finding suggests that chronic mechanical deformation may result in persistent anatomical alterations even after the underlying inflammatory process has been controlled.

While glucocorticoids provided an effective initial response, intravenous methylprednisolone pulse therapy was selected for induction to rapidly alleviate the critical mass effect threatening visual function, in accordance with international consensus recommendations for organ-threatening IgG4-RD (6, 8). Nevertheless, the risk of relapse remains a major concern in the management of IgG4-RD. The recent approval of inebilizumab in Japan (2025) offers a new targeted strategy to maintain remission and reduce steroid dependency. Given the severity of the neurological manifestations and the structural changes observed in this case, long-term maintenance therapy with such B-cell–targeted agents may be considered to prevent disease flares and irreversible damage. This case therefore represents a rare presentation of IgG4-related hypertrophic pachymeningitis characterized by massive dural thickening, extensive white matter lesions, and rectus gyrus herniation. The combination of these findings—particularly chronic herniation into the sella turcica—has not been previously reported. Our observations underscore the clinical importance of recognizing such atypical manifestations and serve as a crucial reminder for neurologists to include IgG4-RD in the differential diagnosis of hypertrophic pachymeningitis, thus ensuring early intervention to prevent irreversible neurological sequelae.

## Data Availability

The original contributions presented in the study are included in the article/supplementary material. Further inquiries can be directed to the corresponding author.
